# Somatic disorders in psychiatric inpatients : Prevalence and associated factors

**DOI:** 10.1192/j.eurpsy.2021.641

**Published:** 2021-08-13

**Authors:** M. Turki, W. Abid, O. Bouzid, S. Ellouze, R. Ouali, N. Halouani, J. Aloulou

**Affiliations:** Psychiatry (b), Hedi Chaker University hospital, sfax, Tunisia

**Keywords:** comorbidity, somatic disorder, psychiatric disorder

## Abstract

**Introduction:**

Elevated prevalence of somatic disorders (SD) in patients with mental diseases is well recognized and studied since latest years. However, their detection remains too late, which darken the prognosis of both diseases, and complicate the therapeutic management.

**Objectives:**

We aimed to determine the prevalence of SD in psychiatric inpatients, and to assess relationships between the two diseases.

**Methods:**

We analyzed retrospectively the medical records of 94 male patients hospitalized for the first time in psychiatry “B” department, Hedi Chaker hospital (Sfax, Tunisia), in the period from January 1^st^ until December 31^st^, 2019.

**Results:**

The mean age of patients was 36.88 years. Among them, 22.3% used cannabis and 37.2% consumed alcohol. Schizophrenia (41,5%) and bipolar disorders (20.2%) were the most common psychiatric diagnoses. During their hospitalization, at list one SD was noted in 53.2%: cardiovascular diseases 21.3% (electrocardiographic anomalies 19,1%); infections 9.6% and hepatic pathologies 8.5 %. The SD was comorbid with psychiatric disease in 90%, and represented a side effect of psychotropics in 10% of patients with SD. Older Patients were more likely to present SD during hospitalization, without a significant association. Patients with schizophrenia were significantly more likely to present infections (p=0.031). Repolarization disorders are more common in patients with cannabis use (p = 0.006).

**Conclusions:**

Our study pointed the high prevalence of SD in patients with mental illnesses, especially in those with schizophrenia and cannabis use. Thus, the somatic assessment should be a systematic practice to identify patients at risk for somatic complications and ensure timely their transfer to a specialized setting.

